# Insights into current coccidioidomycosis therapeutic pathways

**DOI:** 10.1128/aac.01465-25

**Published:** 2025-11-18

**Authors:** Brent Edwards, Rebecca Ripperton, Adeleine Tilley, Daniel B. Chastain, George R. Thompson, Andrés F. Henao-Martínez

**Affiliations:** 1 Department of Medicine, Division of Infectious Diseases, University of Colorado Denver 12226, Aurora, Colorado, USA; 2 Department of Clinical and Administrative Pharmacy, UGA College of Pharmacy 15506, Albany, Georgia, USA; 3 University of California-Davis, Medical Center 21772, Sacramento, California, USA; University of Iowa, Iowa City, Iowa, USA

**Keywords:** Valley fever, coccidioidomycosis, antimicrobial resistance, antifungal therapy, treatment pathways

## Abstract

Coccidioidomycosis is a systemic fungal infection caused by *Coccidioides immitis* and *Coccidioides posadasii*, which are endemic to the Southwestern United States and parts of Mexico, Central, and South America. While oral fluconazole is widely recommended as first-line therapy, treatment failure is not uncommon for severe manifestations. This study aims to characterize antifungal treatment pathways, identify factors associated with treatment switches, and compare outcomes based on treatment courses. A retrospective cohort study was conducted using the TriNetX database with individual de-identified clinical data. Using International Classification of Diseases, Tenth Revision, Clinical Modification codes, we identified patients diagnosed with coccidioidomycosis between January 2010 and March 2024. “Treatment switches” were defined as receiving a different antifungal agent for at least three consecutive days after an initial regimen. Patient demographics, comorbidities, hospitalizations, ICU admissions, and mortality were assessed. A total of 1,909 patients met the inclusion criteria, with 1,581 having complete treatment pathway data. The majority (87.4%) began treatment with fluconazole, followed by amphotericin B (6.5%) and echinocandins (2.3%); 11.2% underwent at least one treatment switch. The most common switches were fluconazole to posaconazole (2.2%) and amphotericin B to fluconazole (1.8%). Coccidioidal meningitis patients switched from fluconazole had increased rates of ICU admission and hospitalization but similar 1-year mortality compared to fluconazole monotherapy. Fluconazole remains the first-line antifungal for coccidioidomycosis, followed by amphotericin B. Most patients required solely fluconazole treatment. While many factors are involved in therapy choice, optimization of therapeutic strategies after initial fluconazole treatment may be necessary for the future, especially for severe disease.

## INTRODUCTION

Coccidioidomycosis (“San Joaquin Valley Fever”) is a systemic fungal infection caused by the dimorphic fungi *Coccidioides immitis* and *Coccidioides posadasii*, which are endemic to regions of the Southwestern United States, Mexico, and parts of South and Central America (1–3). Approximately 273,000 cases of symptomatic coccidioidomycosis were estimated in 2019, with an estimated 23,000 hospitalizations (4). The most common manifestation among symptomatic cases is primary pulmonary coccidioidomycosis, which can present either as focal pneumonia or diffuse reticulonodular pneumonia (5, 6). Dissemination outside of the thorax is less common but may result in devastating sequelae, including coccidioidal meningitis (6). As with other opportunistic infections, individuals with impaired cellular immunity are at heightened risk of severe or disseminated disease, whether from novel or reactivated infection (7, 8).

In 2016*,* the Infectious Diseases Society of America published the *Clinical Practice Guideline for the Treatment of Coccidioidomycosis*, emphasizing oral triazole therapy as the mainstay of first-line therapy (9). Fluconazole is typically preferred due to its bioavailability, cost, and limited propensity for drug-drug interactions. In cases of treatment failure, dose escalation or switching to an alternative therapeutic agent—including other triazoles, polyenes (e.g., intrathecal amphotericin B), and echinocandins—may be considered (9, 10).

Despite these established treatment guidelines, the factors underlying treatment failure are poorly understood, although elevated fluconazole mean inhibitory concentrations (MICs) have been suggested as a possible explanation/contributing factor (10). Furthermore, few data exist on the overall efficacy of other anticoccidioidal regimens, as only a single randomized controlled trial has been performed to date (fluconazole vs itraconazole) (11). Consequently, treatment approaches remain disparate and highly variable between clinicians (9).

This study aims to identify factors associated with treatment switches from the initial antifungal agent to an alternative agent. We examine clinical and demographic characteristics, including significant comorbidities and concurrent opportunistic infections, that may predispose patients to suboptimal outcomes. We also assessed the efficacy of treatment alternatives to fluconazole by evaluating hospitalization rates, ICU admission, 1-year mortality, overall death rates, and survival outcomes. By clarifying the drivers of treatment failure and comparing therapeutic options, we seek to inform evidence-based strategies for managing coccidioidomycosis in the inpatient and outpatient settings.

## MATERIALS AND METHODS

### Data acquisition

We conducted a multicenter retrospective study by querying TriNetX, a global research network (https://trinetx.com/), to identify patients diagnosed with coccidioidomycosis. TriNetX collects data from more than 250 million patients from 250 healthcare organizations across 30 countries, including the United States, mainly large academic medical institutions with inpatient and outpatient services (12). Our group has published several cohort studies using this database (13–20). TriNetX receives inpatient and outpatient data directly from each institution with an average lag time of 1 month. Our data were collected from January 2010 to March 2024.

### Study design and population

To determine the treatment that patients with coccidioidomycosis infections received through the course of their disease, cases and subtypes of coccidioidomycosis were identified using International Classification of Diseases, Tenth Revision, Clinical Modification (ICD-10-CM) codes (Table S1 through S3). Patients may have more than one type of coccidioidomycosis, depending on the severity and progression of their disease. We also collected information regarding patient demographics, their medical comorbidities, associated serology values, and outcomes of interest after 1 year. Comorbidities were specifically identified using ICD-10-CM codes (Table S1 through S3). Patient comorbidities and co-infections were only included if they were diagnosed before the onset of *Coccidioides* diagnosis (non-time-bounded). Laboratory values were determined using LOINC codes. Outcomes assessed included hospitalizations, ICU admissions within 60 days of diagnosis, overall mortality, and 1-year mortality. We obtained 30-days and 1-year mortality. One-year mortality was defined as the number of patients who died within 1 year of coccidioidomycosis diagnosis. Overall mortality refers to the total number of patients who have died since the diagnosis. ICU admissions include certain ICU procedures, including intubation, ventilator management, and ECMO. Hospitalizations and ICU admissions are mutually exclusive.

### Treatment pathways

Specific antifungal medications were identified using RxNorm codes (Table S2). The time course for treatment was defined as 3 months before the initial diagnosis of coccidioidomycosis and up to 6 months after the latest treatment. We excluded combination therapy from the pathway analysis due to its rarity. We captured the recorded treatments 3 months before the diagnosis to ensure that treatments administered before the official diagnosis were included within our analysis. We then analyzed whether prescribed antifungal medications were switched throughout treatment. A treatment line continued until the same antifungal was administered for at least 3 days. A treatment line was defined as the continuous administration of the same antifungal agent for at least 3 consecutive days. A treatment switch was defined as a change to a different antifungal agent, separated from the prior treatment by a minimum gap of 3 dayss. Once each patient’s treatment order was determined, the SunburstR package in R Studio (version 3.6.0) was used to visualize treatment pathways for the entire cohort (21).

### Statistical analysis

Descriptive statistics were presented using means and standard deviations for continuous variables, frequency distributions with proportions, and chi-square analysis for categorical variables.

## RESULTS

Within the defined study period, 1,909 patients were diagnosed with coccidioidomycosis via ICD-10-CM (Table 1). The average age of the cohort was 51.1 (SD ±18.3), and most patients were female. White individuals made up more than half of the cohort, and patients whose race was unknown were the next largest group. Slightly over half of the cases in our cohort were identified within the Western United States. Common comorbid conditions include type 2 diabetes mellitus (T2DM, *n* = 731), chronic kidney disease (CKD, *n* = 590), and unspecified neoplasm (*n* = 479). Other significant comorbidities included opportunistic infections such as candidiasis (*n* = 284), cytomegalovirus (*n* = 137), and cryptococcosis (*n* = 79). Common laboratory trends included mild anemia, lymphopenia (including CD4 counts), and mildly elevated mean lactate dehydrogenase. Approximately a fifth of patients required ICU admission and hospitalization within 60 days of diagnosis, and 11% of patients died within 1 year. The 30-day mortality was 3%.

**TABLE 1:** Characterization of coccidioidomycosis patients, including clinical characteristics and outcomes

Variable	Mean ± SD, *n* (%)
Demographics	*N* = 1,909
Age	51.1 ± 18.3
Sex	
Women	1,176 (63.9)
Men	664 (36.1)
Race	
White	1,053 (55.2)
Unknown	295 (15.5)
Other race	226 (11.8)
Black	196 (10.3)
Asian	87 (4.6)
American Indian	40 (2.1)
Native Hawaiian	12 (0.6)
Ethnicity	
Not Hispanic	1,114 (58.4)
Hispanic	420 (22.0)
Unknown	375 (19.6)
Marital status	
Unknown	1,178 (61.7)
Single	426 (22.3)
Married	305 (16.0)
Region	
West	1,056 (55.3)
South	436 (22.8)
Midwest	190 (10.0)
Northeast	178 (9.3)
Unknown	45 (2.4)
Ex-US	4 (0.2)
Diagnoses	
Comorbidities^*a*^	
Type 2 diabetes mellitus	731 (38.3)
Chronic kidney disease	590 (30.9)
Neoplasm	479 (25.1)
Organ or tissue transplant	360 (18.9)
Heart failure	347 (18.2)
Chronic obstructive pulmonary disease	259 (13.6)
Liver disease	223 (11.7)
Aplastic anemia	220 (11.5)
Inflammatory bowel disease	220 (11.5)
Pulmonary fibrosis	166 (8.7)
Heme malignancy	148 (7.8)
Human immunodeficiency virus	131 (6.9)
Systemic connective tissue diseases	120 (6.3)
Hypogammaglobulinemia	51 (2.7)
Sarcoid	35 (1.8)
Multiple myeloma	23 (1.2)
Rheumatoid arthritis	22 (1.2)
Charlson comorbidity index	0.2 (0.9)
Other opportunistic infections^*a*^	
Candidiasis	284 (14.9)
Cytomegalovirus	137 (7.2)
Cryptococcosis	79 (4.1)
Fusariosis	61 (3.2)
Aspergillosis	58 (3.0)
*Pneumocystis jiroveci* pneumonia	37 (1.9)
Histoplasmosis	25 (1.3)
Blastomycosis	12 (0.6)
Mucormycosis	7 (0.4)
Type of coccidioidomycosis	
Pulmonary coccidioidomycosis	993 (52.0)
Disseminated coccidioidomycosis	290 (15.2)
Acute pulmonary coccidioidomycosis	231 (12.1)
Chronic pulmonary coccidioidomycosis	220 (11.5)
Coccidioidal meningitis	218 (11.4)
Cutaneous coccidioidomycosis	58 (3.0)
Prostatic coccidioidomycosis	4 (0.2)
Labs	Mean (±SD)
White blood cell count	9.7 (7.5)
CD4	349.1 (404.7)
Lymph absolute	32.4 (193.4)
Lymphs (%)	18.6 (15.0)
Hemoglobin	11.7 (2.6)
Hematocrit	39.0 (109.9)
Platelets	255.7 (129.0)
Creatinine	1.4 (1.7)
Alanine transaminase	38.9 (51.2)
Aspartate transferase	45.2 (91.2)
Lactate dehydrogenase	381.0 (229.8)
Ferritin	1,371.9 (4,082.2)
Erythrocyte sedimentation rate	46.5 (33.8)
C-reactive protein	76.2 (88.9)
Procalcitonin	6.2 (33.1)
(1,3)-β-D-glucan	8.3 (31.9)
Outcome	*N* (%)
Intensive care unit (<60 days of diagnosis)	394 (21.4)
Hospitalization (<60 days of diagnosis)	376 (20.5)
Overall death	328 (17.2)
Mortality 1 year	176 (11.0)

^*a*^ Comorbidities and opportunistic infections in this table were identified before the onset of coccidioidomycosis diagnosis.

**Fig 1:**
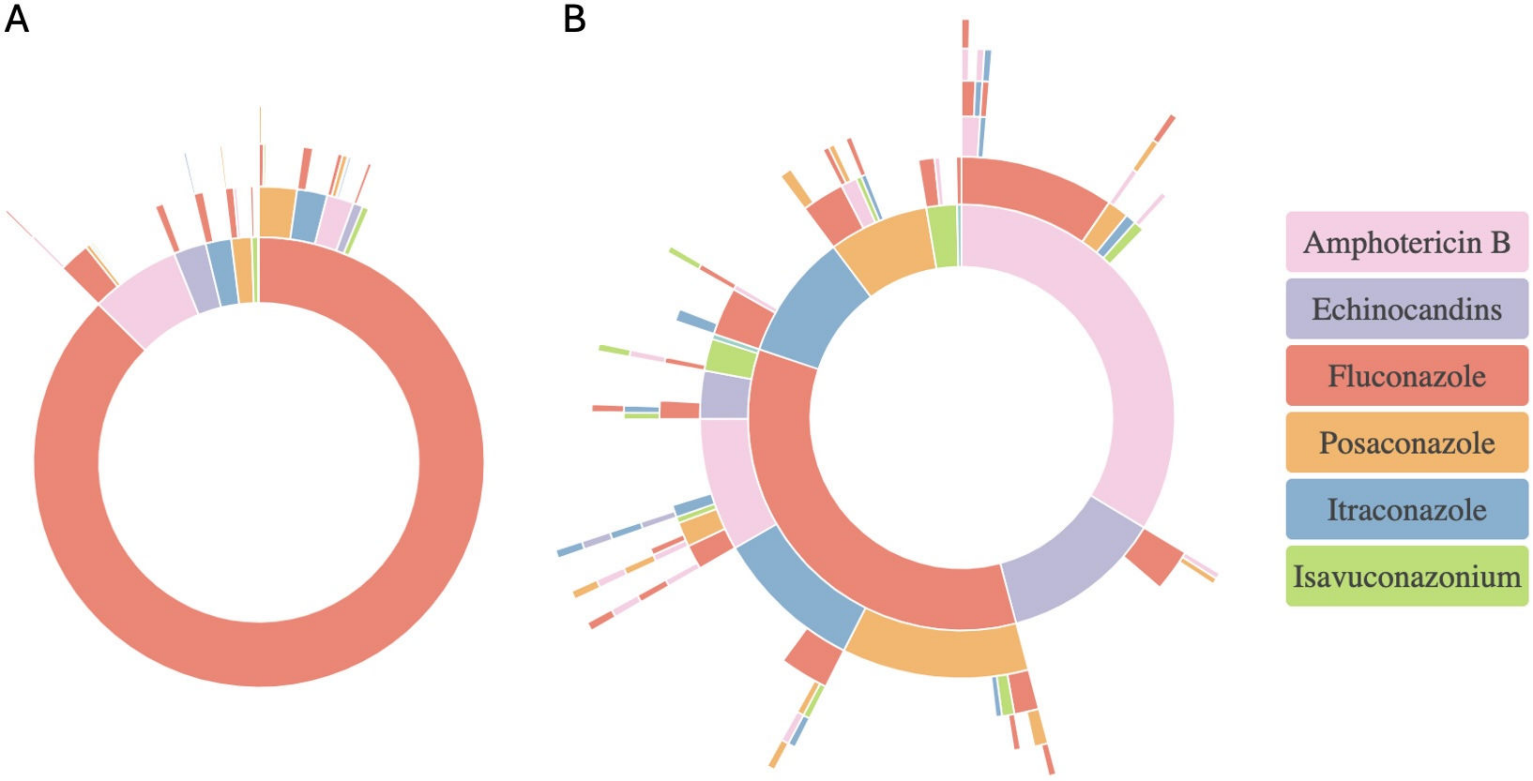
Treatment pathways in coccidioidomycosis. Sunburst diagram of initial treatment choices for coccidioidomycosis. (**A**) Treatment pathways were analyzed for the 1,581 patients with available treatment data who were diagnosed with coccidioidomycosis. (**B**) A subset of the treatment pathway analysis, excluding patients with fluconazole monotherapy, to better visualize other treatments used. Each ring represents a line of treatment. The center of the ring is the initial treatment, while subsequent rings represent switches. A treatment line was defined as the continuous administration of the same antifungal agent for at least 3 consecutive days. A treatment switch was defined as a change to a different antifungal agent, separated from the prior treatment by a minimum gap of 3 days. Combination therapies were excluded.

Of the 1,909 patients in the initial cohort diagnosed with coccidioidomycosis, 1,581 had associated information regarding their treatment pathway. We excluded the 328 patients without an available treatment history from the analysis. Additionally, only six patients were found to have received combination therapy, defined as receiving two different antifungals within 3 days of each other. No patients were on continuous combination therapy (both medications >5 days), yet they were still excluded from the study. Fluconazole was overwhelmingly the initial antifungal used for coccidioidomycosis (Fig. 1A), followed by amphotericin B (lipid complex and lisosomal formulation) and echinocandins. Most patients received only a single antifungal agent throughout their treatment, with 11.2% undergoing treatment switches (Fig. 1A). Only seven patients in the cohort received more than two different azoles during treatment. The most common initial switches were fluconazole to posaconazole (Fig. 1B), followed by amphotericin B to fluconazole (likely representing step-down therapy in those with severe disease), and fluconazole to itraconazole.

**Fig 2:**
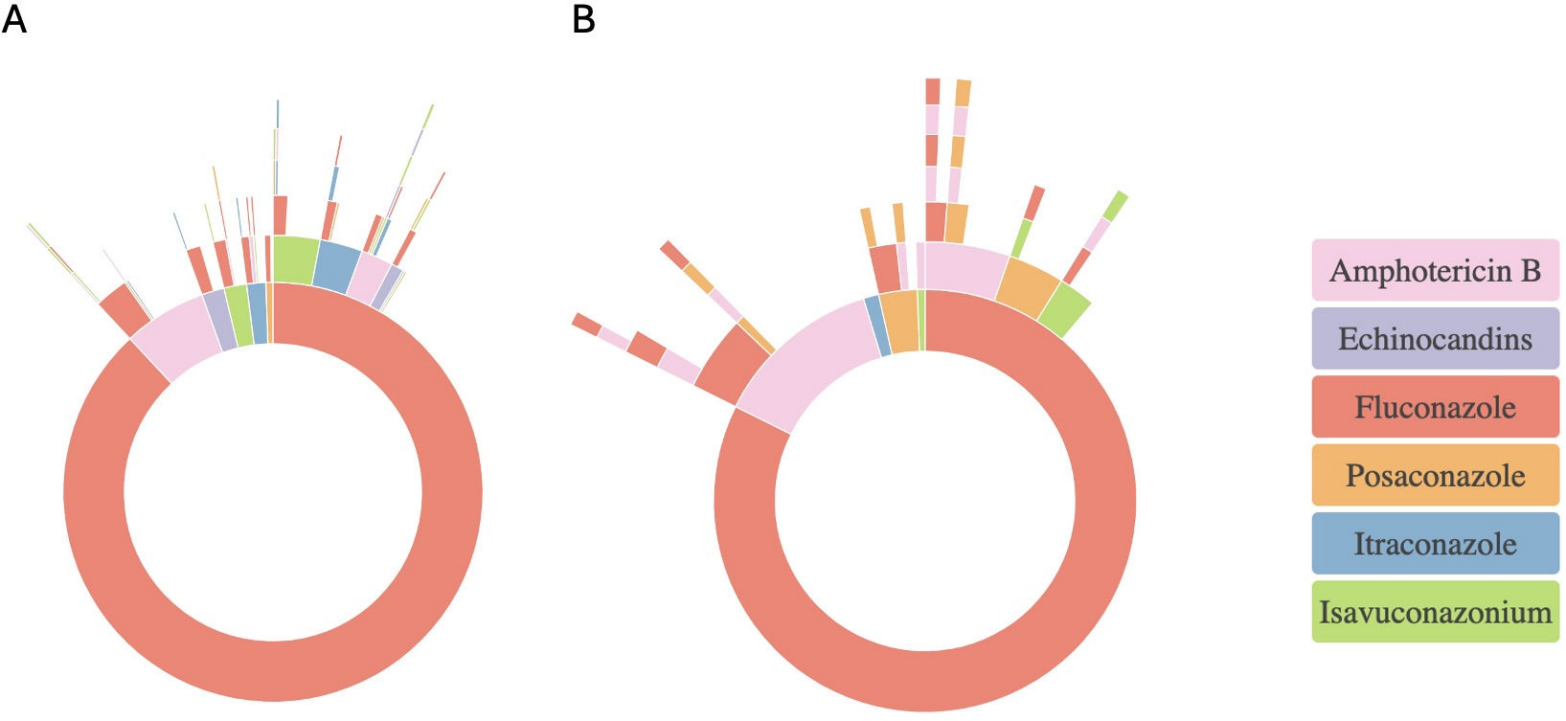
Coccidioidomycosis treatment pathways represented as sunburst diagrams for subtypes of *Coccidioides* infection. (**A**) Pulmonary coccidioidomycosis. (**B**) Coccidioidal meningitis. Each ring represents a line of treatment. The center of the ring is the initial treatment, while subsequent rings represent switches. The time course for treatment was defined as 3 months before the initial diagnosis of coccidioidomycosis and up to 6 months after the latest treatment. A treatment line was defined as the continuous administration of the same antifungal agent for at least 3 consecutive days. A treatment switch was defined as a change to a different antifungal agent, separated from the prior treatment by a minimum gap of 3 days. Combination therapies were excluded.

We investigated whether treatment pathways differed for those diagnosed with different manifestations of *Coccidioides* spp. infections. For those with treatment pathway information available, the most common presentation of coccidioidomycosis was pulmonary infection, followed by disseminated coccidioidomycosis, coccidioidal meningitis, and cutaneous coccidioidomycosis. For this analysis, we excluded *Coccidioides* spp. infection subtypes designated as “unspecified” or “other” coccidioidomycosis infections. Those with pulmonary *Coccidioides* spp. had a similar treatment pathway distribution as the general cohort, with most patients receiving fluconazole as the initial treatment (Fig. 2A). The most common switches were fluconazole to itraconazole (Fig. 2A), fluconazole to posaconazole, and amphotericin B to fluconazole. The most likely treatment switch for cutaneous coccidioidomycosis was from fluconazole to itraconazole (Fig. S1A), while the most common switch for disseminated coccidioidomycosis was from fluconazole to posaconazole, followed by fluconazole to amphotericin B (Fig. S1B).

Next, we investigated whether there were any differences in comorbidities, demographics, or outcomes for pulmonary infections treated solely with fluconazole compared to those who switched from fluconazole as the initial agent. For pulmonary infections, we identified 658 patients who received fluconazole as their initial treatment, with 592 receiving only fluconazole and 66 undergoing a treatment switch. Overall, the demographics of the fluconazole-only treatment group and the patients who switched from fluconazole were very similar (Table S4). There were no significant differences in race, ethnicity, or region between the fluconazole-only patients and the group switched from fluconazole to an alternative agent. For the most prevalent comorbidities, including T2DM, CKD, neoplasm, and transplant, there was no significant difference between the fluconazole-only and the fluconazole switch group. Patients who switched from fluconazole had an increased rate of aplastic anemia (*P* < 0.01) and hypogammaglobulinemia (*P* < 0.05) compared to the fluconazole-only group. There were no significant differences in opportunistic infections between the treatment groups. There was also no significant difference in ICU admissions, hospitalizations, overall death, or 1-year mortality between the fluconazole-only and switch groups.

Due to the specific challenges posed by CNS coccidioidomycosis, we investigated whether patients switched from fluconazole had underlying differences in demographics, comorbidities, or outcomes compared to those treated with only fluconazole. There were no significant differences in race or region between fluconazole-only patients compared to patients who switched from fluconazole within the meningococcal coccidioidomycosis subgroup (Table 2). For patients with meningeal *Coccidioides* spp., the most common switches were from fluconazole to amphotericin B, amphotericin B to fluconazole, and fluconazole to posaconazole (Fig. 2B). There was a significantly lower number of patients with unknown ethnicity in the fluconazole switch group than in the fluconazole-only group. There were no underlying differences in comorbidities between the groups. Within this subgroup, patients who switched from fluconazole to other antifungal agents were significantly more likely to be admitted to the ICU (*P* = 0.02) or hospitalized (*P* = 0.01) than patients receiving only fluconazole throughout the course of their treatment. Despite increased hospital admissions, the overall death rate and 1-year mortality did not differ between the two treatment groups.

**TABLE 2:** Characterization of demographics, comorbidities, and outcomes for patients diagnosed with coccidioidal meningitis who received fluconazole monotherapy and those who switched from fluconazole

	Fluconazole only (*N* = 121)	Switched from fluconazole (*N* = 19)	*P* value
Demographics			
Age (±SD)	47.8 (± 17.4)	30.4 (± 17.0)	<0.01
Sex			
Men	87 (71.9%)	16 (84.2%)	0.39
Women	28 (23.1%)	3 (15.8%)	0.67
Race			
White	50 (41.3%)	10 (52.6%)	0.50
Unknown	29 (24.0%)	2 (10.5%)	0.31
Other race	16 (13.2%)	2 (10.5%)	1.00
Black	13 (10.7%)	3 (15.8%)	0.80
Asian	6 (5.0%)	2 (10.5%)	0.66
American Indian	5 (4.1%)	0 (0.0%)	0.81
Native Hawaiian	1 (0.8%)	0 (0.0%)	1.00
Ethnicity			
Not Hispanic	58 (47.9%)	11 (57.9%)	0.58
Unknown	37 (30.6%)	1 (5.3%)	0.04
Hispanic	25 (20.7%)	7 (36.8%)	0.20
Marital status			
Unknown	85 (70.2%)	10 (52.6%)	0.21
Single	21 (17.4%)	8 (42.1%)	0.03
Married	14 (11.6%)	1 (5.3%)	0.67
Region			
West	72 (59.5%)	13 (68.4%)	0.63
South	22 (18.2%)	3 (15.8%)	1.00
Northeast	14 (11.6%)	1 (5.3%)	0.67
Midwest	9 (7.4%)	2 (10.5%)	0.99
Unknown	3 (2.5%)	0 (0.0%)	1.00
Comorbidities			
Type 2 diabetes mellitus	23 (19.0%)	1 (5.3%)	0.25
Human immunodeficiency virus	15 (12.4%)	2 (10.5%)	1.00
Neoplasm	11 (9.1%)	2 (10.5%)	1.00
Chronic kidney disease	9 (7.4%)	0 (0.0%)	0.47
Organ or tissue transplant	3 (2.5%)	0 (0.0%)	1.00
Inflammatory bowel disease	2 (1.7%)	2 (10.5%)	0.16
Liver disease	2 (1.7%)	0 (0.0%)	1.00
Aplastic anemia	2 (1.7%)	0 (0.0%)	1.00
Heme malignancy	2 (1.7%)	0 (0.0%)	1.00
Systemic connective tissue disorders	2 (1.7%)	0 (0.0%)	1.00
Sarcoidosis	2 (1.7%)	0 (0.0%)	1.00
Unspecified immunodeficiency	2 (1.7%)	0 (0.0%)	1.00
Heart failure	1 (0.8%)	0 (0.0%)	1.00
Chronic obstructive pulmonary disease	1 (0.8%)	0 (0.0%)	1.00
Rheumatoid arthritis	1 (0.8%)	0 (0.0%)	1.00
Pulmonary fibrosis	0 (0.0%)	0 (0.0%)	1.00
Hypogammaglobulinemia	0 (0.0%)	0 (0.0%)	1.00
Multiple myeloma	0 (0.0%)	0 (0.0%)	1.00
Common variable immunodeficiency disorder	0 (0.0%)	0 (0.0%)	1.00
Other opportunistic infections			
Candidiasis	3 (2.5%)	0 (0.0%)	1.00
Cytomegalovirus	2 (1.7%)	0 (0.0%)	1.00
Cryptococcosis	5 (4.1%)	0 (0.0%)	0.81
Fusariosis	2 (1.7%)	0 (0.0%)	1.00
*Pneumocystis jiroveci* pneumonia	1 (0.8%)	0 (0.0%)	1.00
Aspergillosis	0 (0.0%)	0 (0.0%)	1.00
Histoplasmosis	0 (0.0%)	0 (0.0%)	1.00
Blastomycosis	0 (0.0%)	0 (0.0%)	1.00
Mucormycosis	0 (0.0%)	0 (0.0%)	1.00
Outcomes			
Intensive care unit	8 (6.6%)	5 (26.3%)	0.02
Hospitalization	7 (5.8%)	5 (26.3%)	0.01
Overall death	17 (14.0%)	2 (10.5%)	0.95
Mortality 1 year	10 (8.3%)	2 (10.5%)	1.00

## DISCUSSION

In this study, we investigated the treatment pathways for a cohort of patients identified to have coccidioidomycosis. We found that 87.4% of patients received fluconazole as the initial treatment. This finding is consistent with fluconazole being the mainstay of therapy for decades (9, 22). Most patients received fluconazole monotherapy as an initial agent, suggesting successful infection management following first-line treatment. The widespread use of fluconazole has been attributed both to its efficacy in less severe cases and overall tolerability (23). Side effects of fluconazole have a relatively low incidence and are typically mild (24), which may explain its use as a frequent initial agent in our study. While symptoms are generally mild, other studies have found that over half of the patients studied experienced adverse effects from fluconazole therapy longer than 28 days (23), which may provide an explanation for treatment switches from fluconazole in our study.

Another potential explanation for fluconazole treatment predominance is related to its ease of administration as an oral medication. In contrast, other treatments, such as amphotericin B, are often administered intravenously or even intrathecally, which may lead to decreased usage, especially in the outpatient setting (25–27). Other potential obstacles to the use of different medications, leading to the high prevalence of fluconazole use, may involve health system logistics. For instance, mold-active triazoles may require prior authorization and are challenging to obtain for most patients with coccidioidomycosis. The choice of fluconazole compared to other triazoles may also be influenced by its lower potential for drug-drug interactions and decreased variability in absorption, particularly compared to itraconazole, which can be affected by diet (9). The frequent selection of fluconazole as the initial antifungal in coccidioidomycosis treatment may be due to many factors, including high efficacy, low rate of side effects, low cost, drug-drug interactions, high oral bioavailability, linear kinetics, and ease of administration.

To gain insight into how the severity of disease impacts *Coccidioides* spp. treatment, we conducted a subgroup analysis of coccidioidal meningitis cases within our cohort. We found that 82.4% of coccidioidal meningitis cases were treated initially with fluconazole, with fluconazole being the sole agent for most cases (Fig. 2B). This is supported by previous findings that fluconazole is sufficient for treating coccidioidal meningitis in most cases (28). In our study, those who switched from fluconazole to other treatments may represent the worsening of severe disease due to fluconazole treatment failure, as observed in other studies of coccidioidal meningitis (29, 30). Fluconazole failure in coccidioidal meningitis has been found to occur in up to one-third of patients (30). While our rate of fluconazole switching in coccidioidal meningitis patients was substantially lower at 15%, our study cannot determine whether treatment switches are attributed to fluconazole failure due to the retrospective study design. The treatment switches observed in our study for coccidioidal meningitis could be related to the increased severity of meningeal infections or decreased antifungal bioavailability in the CNS (30). The worsening of disease in some cases despite fluconazole treatment suggests that fluconazole monotherapy may be insufficient for severe meningeal disease (31). This aligns with recent findings showing that, although azoles remain the primary agents used for CNS coccidioidomycosis, outcomes in these cases are often poor, with high hospitalization costs and frequent rebound admissions (31).

While fluconazole failure in cases of severe disease explains treatment switches, another aspect that must be considered is the pharmacokinetic and pharmacodynamic factors within the CNS that may make treatment suboptimal. Fluconazole may be an effective therapeutic option during the therapy of non-CNS-infected patients due to its high oral bioavailability and linear kinetics. However, the CNS offers numerous barriers to drug penetration (32). Fluconazole cerebrospinal fluid levels are approximately 50% of plasma at this site, and pharmacokinetic and pharmacodynamic parameters may be subtherapeutic. Studies of *in vitro Coccidioides* spp. isolates have found a decreased fluconazole susceptibility, with more than one-third of isolates developing high MICs (31–33). Other studies have found that fluconazole doses must often be increased from guidelines to see a clinical effect (31) and often require close monitoring (34). Together, these studies show that the development of high MIC in *Coccidioides* spp. may precipitate fluconazole failure due to antifungal resistance and thus lead to escalation of therapy via increasing doses of current therapy or switching treatments. Our study found that almost half of patients with CNS disease switched from fluconazole to amphotericin B as a second-line therapy as opposed to other azoles. There are a number of explanations for treatment switches in these scenarios, one being fluconazole failure. Other aspects to consider are that initial drug selection may be the result of empiric therapy, and the switch to amphotericin B is the result of discovery of CNS disease. The design of our study is unable to distinguish the causative factors for treatment switches, but treatment failure is an important factor to consider due to the severity of CNS infections.

Severe coccidioidomycosis has been associated with those who are immunocompromised and those who develop severe infections such as coccidioidal meningitis (3, 9, 35). Clinical predictors of fluconazole failure in coccidioidal meningitis include the length of initial neurologic symptoms and the presence of encephalopathy on initial presentation (29). For infections refractory to fluconazole treatment, case reports have found liposomal amphotericin B successfully to treat individuals with coccidioidal meningitis (26). While our study is unable to infer causation for treatment switches, we found amphotericin B was the second agent used after fluconazole in patients with coccidioidal meningitis. This is distinct from other findings, which showed the most frequent second-line therapies in coccidioidal meningitis were triazoles, such as posaconazole and itraconazole (29). While our study is unable to deduce the reason for treatment failure, other studies have shown that triazoles such as isavuconazole have shown promising results in chronic *Coccidioides* spp. infections, particularly for skin and soft tissue, and pulmonary manifestations, yet there was still frequent treatment failure for coccidioidal meningitis (36). Several novel therapies have also succeeded *in vitro* against *Coccidioides* or other invasive fungi (37, 38). This includes olorofim, a dihydroorotate dehydrogenase inhibitor, which has been effective against all *Coccidioides* strains tested *in vitro* at much lower concentrations than fluconazole (39). Another agent, rezafungin, is more effective against *Candida* isolates than fluconazole after *in vitro* testing (40). Our study shows that fluconazole is still relied on for many cases of coccidioidal meningitis, which may be impacted by the development of high MICs to fluconazole, providing a compelling explanation for potential treatment switches. Still, it does not explain all factors contributing to therapy selection, and novel therapeutics offer potential treatments in fluconazole-refractory cases.

The limitations of this study primarily focus on the retrospective nature of the study and the limited information available via the TriNetX database analysis. While the data on TriNetX aggregate data from many hospitals and medical systems, we are unable to determine the clinical rationale for treatment switches based on the data set and its retrospective nature. Similarly, the reason for the initial antifungal agent selection or specific amphotericin formulations was unavailable. This leaves us to postulate on treatment decisions that rely on many factors, including inpatient vs outpatient status, severity of disease, immunocompetence of host, antifungal side effect profiles, or empiric treatment as opposed to targeted therapy, among others. Additionally, all diagnoses were determined by ICD-10-CM codes instead of laboratory testing, which can introduce reliability issues related to incomplete data or empiric therapies and are limitations inherent to large data sets using ICD coding.

Additionally, this study did not factor antifungal dosing into treatment switches, so we are unable to observe whether therapy was escalated within a common medication. This study also did not analyze whether *Coccidioides* spp. infection was the primary reason for encountering the medical system.

Another factor that could influence the results of this study was the inclusion of co-infections from other comorbidities. While part of the cohort had a history of candidal and cryptococcal infections, this does not indicate that patients were experiencing co-infection with *Coccidioides* and other opportunistic infections. Isolated case reports of *Coccidioides* and *Cryptococcus* co-infections have been reported, yet the overall prevalence of this occurrence is rare (41).

This study does not examine the specific antifungal dosages administered to each patient. To better understand how disease severity influences treatment decisions, future research should explore whether variations in treatment dosages correlate with treatment modifications or specific disease subtypes. Additionally, assessing how particular comorbidities or immunodeficiencies impact treatment strategies could offer valuable clinical insights for managing affected individuals.

### Conclusion

Fluconazole monotherapy was the primary initial treatment for *Coccidioides* spp. infections. Switches were infrequent; however, amphotericin B was a common alternative when they occurred. In instances of treatment switching, it is unclear whether switches were made due to increased disease severity, development of fluconazole resistance, or an alternative reason. Beyond monotherapy with fluconazole, treatment pathways remain clinically heterogeneous, suggesting the need for more explicit clinical guidance in managing severe or refractory disease.

## SUPPLEMENTAL MATERIAL

Supplemental Material aac.01465-25-s0001.docx Tables S1 to S4; Fig. S1.

ASM does not own the copyrights to Supplemental Material that may be linked to, or accessed through, an article. The authors have granted ASM a non-exclusive, world-wide license to publish the Supplemental Material files. Please contact the corresponding author directly for reuse.
