# Supplementary material for: Insights into current coccidioidomycosis therapeutic pathways
Source: Antimicrob Agents Chemother. 2025 Nov 18;69(12):e01465-25. doi: 10.1128/aac.01465-25 (PMC12691651; doi:10.1128/aac.01465-25)
Supplement: Supplemental Material — Tables S1 to S4; Fig. S1. [file aac.01465-25-s0001.docx]

**Supplementary Online Content**

**Table S1. ICD-10 codes used to identify cases of coccidioidomycosis and the subtype of infection.**

**Table S2. RxNorm codes used to identify which antifungal was used in the treatment of coccidioidomycosis.**

**Table S3. International Classification of Diseases, Tenth Revision, Clinical Modification (ICD-10-CM) diagnosis codes for underlying comorbidities.**

**Table S4.** **Characterization of demographics, comorbidities, and outcomes for patients diagnosed with pulmonary coccidioidomycosis who received fluconazole monotherapy and those who switched from fluconazole.**

**Figure S1. Treatment pathways for disseminated and cutaneous coccidioidomycosis patients.**

**Table S1. ICD-10 codes used to identify cases of coccidioidomycosis and the subtype of infection.**

| Type of Cocci | ICD-10-Code |
| --- | --- |
| Coccidioidomycosis | B38 |
| Acute pulmonary coccidioidomycosis | B38.0 |
| Chronic pulmonary coccidioidomycosis | B38.1 |
| Pulmonary coccidioidomycosis, unspecified | B38.2 |
| Cutaneous coccidioidomycosis | B38.3 |
| Coccidioidomycosis meningitis | B38.4 |
| Disseminated coccidioidomycosis | B38.7 |
| Other forms of coccidioidomycosis | B38.8 |
| Prostatic coccidioidomycosis | B38.81 |
| Other forms of coccidioidomycosis | B38.89 |
| Coccidioidomycosis, unspecified | B38.9 |

**Table S2. RxNorm codes used to identify which antifungal was used in the treatment of coccidioidomycosis.**

| Antifungal | RxNorm |
| --- | --- |
| fluconazole | 4450 |
| isavuconazonium | 1608322 |
| amphotericin B | 732 |
| amphotericin | 42527 |
| posaconazole | 282446 |
| itraconazole | 28031 |
| ibrexafungerp | 2560213 |
| caspofungin | 140108 |
| anidulafungin | 341018 |
| micafungin | 325887 |

**Table S3. International Classification of Diseases, Tenth Reviision, Clinical Modification (ICD-10-CM) diagnosis codes for underlying comorbidities.**

| Type 2 Diabetes Mellitus | E11 |
| --- | --- |
| Chronic Kidney Disease | N18 |
| Neoplasm | C00-D49 |
| Transplant | Z94 |
| Heart Failure | I50 |
| Chronic Obstructive Pulmonary Disease | J44 |
| Liver Disease | K70-K75 |
| Aplastic | D60, D61 |
| Inflammatory Bowel Disease | K50-K52 |
| Pulmonary Fibrosis | J84 |
| Heme Malignancy | C81-C86, C88, C90-C96 |
| Human Immunodeficiency Virus | B20 |
| Systemic Connective Tissue Disorder | M35, M36 |
| Hypogammaglobulinemia | D80.0, D80.1 |
| Sarcoid | D86 |
| Multiple Myeloma | C90 |
| RA | M05, M06 |
| Unspecified Immunodeficiency | B84.9 |
| Common Variable Immunodeficiency Disorder | B83.9 |
| Candidiasis | B37 |
| CMV | B25 |
| Cryptococcosis | B45 |
| Fusariosis | B48.8 |
| Aspergillosis | B44 |
| PJP | B59 |
| Histoplasmosis | B39 |
| Blastomycosis | B40 |
| Mucormycosis | B46 |

**Table S4.** **Characterization of demographics, comorbidities, and outcomes for patients diagnosed with pulmonary coccidioidomycosis who received fluconazole monotherapy and those who switched from fluconazole.**

|  | Fluconazole Only (N = 592) | Switched from Fluconazole (N = 66) | p value |
| --- | --- | --- | --- |
| **Demographics** |  |  |  |
| Age (± SD) | 52.8 (± 17.5) | 46.4 (± 18.4) | < 0.01 |
| Sex | | |  |
| Women | 199 (33.6%) | 19 (28.8%) | 0.51 |
| Men | 370 (62.5%) | 42 (63.6) | 0.96 |
| Race | | |  |
| White | 332 (56.1%) | 35 (53.0%) | 0.73 |
| Unknown | 105 (17.7%) | 11 (16.7%) | 0.96 |
| Other race | 65 (11.0%) | 8 (12.1%) | 0.94 |
| Black | 41 (6.9%) | 7 (10.6%) | 0.20 |
| Asian | 32 (5.4%) | 4 (6.1%) | 1.00 |
| American Indian | 14 (2.3%) | 0 (0%) | 0.42 |
| Native Hawaiian | 3 (0.5%) | 1 (1.5%) | 0.87 |
| Ethnicity | | |  |
| Not Hispanic | 318 (53.7%) | 41 (62.1%) | 0.24 |
| Hispanic | 138 (23.3%) | 13 (19.7%) | 0.61 |
| Unknown | 136 (23.0%) | 12 (18.2%) | 0.47 |
| Marital Status | | |  |
| Unknown | 371 (62.7%) | 31 (47.0%) | 0.02 |
| Single | 117 (19.8%) | 25 (37.9%) | 0.001 |
| Married | 104 (17.6%) | 10 (15.2%) | 0.75 |
| Region | | |  |
| West | 334 (56.4%) | 37 (56.1%) | 1.00 |
| South | 120 (20.3%) | 19 (28.8%) | 0.15 |
| Midwest | 57 (9.6%) | 7 (10.6%) | 0.97 |
| Northeast | 48 (8.1%) | 2 (3.0%) | 0.22 |
| Unknown | 31 (5.2%) | 1 (1.5%) | 0.30 |
| Ex-US | 2 (0.3%) | 0 (0%) | 1.00 |
|  |  |  |  |
| **Co-morbidities** |  |  |  |
| Type 2 Diabetes mellitus | 197 (33.3%) | 20 (30.3%) | 0.73 |
| Chronic Kidney Disease | 127 (21.5%) | 16 (24.2%) | 0.72 |
| Neoplasm | 167 (28.2%) | 15 (22.7%) | 0.42 |
| Heart Failure | 69 (11.7%) | 5 (7.6%) | 0.43 |
| Transplant | 63 (10.6%) | 10 (15.2%) | 0.37 |
| Chronic Obstructive Pulmonary Disease | 62 (10.5%) | 4 (6.1%) | 0.36 |
| Liver Disease | 46 (7.8%) | 9 (13.6%) | 0.16 |
| Pulmonary Fibrosis | 41 (6.9%) | 6 (9.1%) | 0.69 |
| Unspecified Immunodeficiency | 33 (5.6%) | 4 (6.1%) | 1 |
| Heme Malignancy | 30 (5.1%) | 3 (4.5%) | 1 |
| Inflammatory Bowel Disease | 24 (4.1%) | 5 (7.6%) | 0.31 |
| Human Immunodeficiency Virus | 21 (3.5%) | 3 (4.5%) | 0.95 |
| Aplastic | 19 (3.2%) | 8 (12.1%) | 0.002 |
| Rheumatoid Arthritis | 19 (3.2%) | 2 (3.0%) | 1 |
| Multiple Myeloma | 8 (1.4%) | 0 (0%) | 0.72 |
| Systemic Connective Tissue Disorder | 6 (1.0%) | 1 (1.5%) | 1 |
| Hypogammaglobulinemia | 5 (0.8%) | 3 (4.5%) | 0.04 |
| Sarcoid | 5 (0.8%) | 0 (0%) | 0.99 |
| Common Variable Immunodeficiency Disorder | 0 (0%) | 0 (0%) | 1 |
|  |  |  |  |
| **Other Opportunistic Infections** |  |  |  |
| Candidiasis | 32 (5.4%) | 4 (6.1%) | 1 |
| CMV | 11 (1.9%) | 4 (6.1%) | 0.08 |
| Cryptococcosis | 12 (2.0%) | 2 (3.0%) | 0.93 |
| Aspergillosis | 8 (1.4%) | 2 (3.0%) | 0.6 |
| Fusariosis | 7 (1.2%) | 0 (0%) | 0.8 |
| PJP | 6 (1.0%) | 1 (1.5%) | 1 |
| Histoplasmosis | 4 (0.7%) | 0 (0%) | 1 |
| Blastomycosis | 2 (0.3%) | 1 (1.5%) | 0.7 |
| Mucormycosis | 1 (0.2%) | 0 (0%) | 1 |
|  |  |  |  |
| **Outcomes** |  |  |  |
| ICU | 58 (9.8%) | 11 (16.7%) | 0.13 |
| Hospitalization | 57 (9.6%) | 10 (15.2%) | 0.23 |
| Overall death | 83 (14.0%) | 10 (15.2%) | 0.95 |
| Mortality 1-year | 58 (9.8%) | 9 (13.6%) | 0.45 |

**Fig S1.** Coccidioidomycosis treatment pathways represented as Sunburst diagrams for subtypes of *Coccidoides* infection. **A.** Cutaneous coccidioidomycosis. **B.** Disseminated coccidioidomycosis. Each ring represents a line of treatment. The center of the ring is the initial treatment, while subsequent rings represent switches. Antifungal treatment initiation was defined as receiving an antifungal within 3 days of coccidioidomycosis diagnosis. A treatment line continued as long as the same antifungal was administered for at least 3 days. A treatment switch occurred when a patient changed to a different antifungal, with a minimum 3-day gap between the prior and new treatment lines. Combination therapies were excluded.
